# Hybrid cooperative complex treatment is associated with reduced facial pore size in a pilot study

**DOI:** 10.1038/s41598-026-56454-0

**Published:** 2026-06-12

**Authors:** Erik Koppert, Roya Zarmehr Zamin, Suyeon Lee, Kyu-Ho Yi

**Affiliations:** 1 Department of Surgery, Epworth Hawthorn and Epworth Eastern Private Hospitals, Melbourne, VIC Australia; 2 Dentofaces Clinic, Dubai, UAE; 3 Medical Research Inc., Seoul, Korea; 4 You and I Clinic, Seoul, Republic of Korea; 5 Division in Anatomy & Developmental Biology, Department of Oral Biology, Yonsei University College of Dentistry, 50-1 Yonsei-ro, Seodaemun-gu, Seoul, 03722 Korea

**Keywords:** Hyaluronic acid, Skin aging, Pores, Facial skin, Bioremodeling agents, Health care, Medical research

## Abstract

Enlarged facial pores are a common aesthetic concern associated with aging and reduced dermal support. Hybrid Cooperative Complex (HCC), a stabilized hyaluronic acid formulation, has been proposed to improve skin quality through bioremodeling. This prospective observational pilot study evaluated changes in facial pore size after treatment with Hybrid Cooperative Complex (HCC) using standardized photography, clinical assessment, and validated rating scales. Ten healthy adult participants received two treatment sessions according to the Bio Aesthetic Points protocol, and outcomes were assessed at baseline and on Days 30, 60, 120, and 180. Visible improvement in facial pore size and skin texture was observed after treatment. Peak pore refinement was noted at Day 120 in most participants, while partial regression was observed in many cases by Day 180, although skin quality remained improved compared with baseline. HCC may be associated with visible improvement in facial pore size and skin quality in this pilot cohort. Changes in pore morphology may represent a practical clinical marker for estimating maintenance treatment timing, although larger controlled studies are needed.

## Introduction

Hybrid cooperative complex (HCC) is designed for individuals experiencing early to moderate signs of skin aging, including loss of elasticity, hydration, and firmness. With advancing age, declining levels of collagen, elastin, and endogenous hyaluronic acid (HA) contribute to structural and functional alterations in the dermis, resulting in compromised barrier function, dehydration, wrinkling, sagging, and enlargement of facial pores^1^.

HCC represents an injectable bioremodeling agent consisting of high- and low-molecular-weight hyaluronic acid (H-HA and L-HA) in a stabilized hybrid cooperative complex. This formulation has been reported to improve skin hydration, tone, elasticity, and texture through dermal bioremodeling mechanisms^2^.

Although the clinical effects of HCC on skin quality have been described, its specific impact on facial pore size, a highly visible and psychologically relevant aesthetic feature, has not been specifically investigated. Previous real-world clinical experience reported that HCC administered using the standardized Bio Aesthetic Points (BAP) technique promoted visible dermal bioremodeling, improved elasticity, and skin tone enhancement^3^.

At the cellular level, Stellavato et al. demonstrated that hybrid cooperative complexes of hyaluronic acid may reactivate fibroblast proliferation and extracellular matrix turnover while maintaining low inflammatory signaling compared with crosslinked gels^4^. These findings provide a biological rationale for the potential dermal remodeling and pore-refining effects hypothesized in the current study.

Moreover, Kim et al. reported that pore volume, rather than pore count, is most strongly correlated with visual assessment of skin pores, supporting the rationale for using pore morphology as a clinically relevant endpoint in aesthetic studies^5^.

HCC differs from traditional fillers in that it is intended to promote pan-dermal remodeling and hydration rather than localized volumization. This distinction supports the design of the present investigation, which focuses on visible changes in pore morphology rather than filling effects^6^.

This pilot study was not intended to evaluate general skin quality alone, but specifically to examine changes in facial pore morphology following HCC treatment. The study was inspired by clinical observations, with the central hypothesis that changes in pore size may serve as a practical, visible marker for determining the optimal timing of maintenance treatments.

IBSA currently recommends a maintenance interval of 2–6 months after an initial two-treatment course spaced four weeks apart^7^. However, clinical experience suggests that pore morphology could help refine this interval based on individualized treatment response.

This study therefore aimed to observe pore size evolution over 180 days as a preliminary investigation of facial pore size as a treatment endpoint for HCC.

## Methods

### Study design and participants

This independent, prospective observational pilot study enrolled ten healthy adult participants (nine females and one male; age range, 32–52 years; mean age, 40.7 years) presenting with visible facial pores and early signs of facial skin aging. Exclusion criteria included aesthetic procedures within the preceding 6 months, active dermatologic disease, infection, pregnancy, or hypersensitivity to hyaluronic acid. No participants received any additional cosmetic treatment during the study period.

Participant demographic characteristics are summarized in Table 1.

**Table 1:** Demographic characteristics of study participants.

Patient ID	Age, years	Sex
P01	42	Female
P02	34	Female
P03	37	Female
P04	32	Male
P05	51	Female
P06	47	Female
P07	36	Female
P08	44	Female
P09	32	Female
P10	52	Female

All methods were carried out in accordance with relevant guidelines and regulations. The study protocol was approved by the Public Institutional Review Board of the Republic of Korea (Approval No. P01-202509-01-018). Written informed consent was obtained from all participants prior to inclusion in the study, including consent for the use of clinical photographs for publication purposes.

### Treatment protocol

All participants received two sessions of HCC (IBSA Farmaceutici, Italy) administered using the Bio Aesthetic Points technique, with five standardized injection sites per hemiface: zygomatic prominence, nasal base, tragus region, chin, and mandibular angle. Each point received 0.2 mL of product at a superficial subcutaneous depth using a 29-gauge needle. The second session was performed 30 days after the first. All procedures were performed by the same experienced injector under aseptic conditions. No topical anesthesia was used, and all procedures were well tolerated without adverse events.

### Photography and clinical evaluation

Standardized photographic documentation was obtained at baseline and on Days 30, 60, 120, and 180 using identical lighting, positioning, and camera settings. Facial pores were evaluated by direct clinical inspection and high-resolution photographic comparison, with particular attention to the malar and submalar regions.

### Outcome measures

Pore size and global skin quality were evaluated using the Scientific Assessment Scale of Skin Quality (SASSQ)^8^. Aesthetic improvement was assessed using the Global Aesthetic Improvement Scale by both the investigator (GAIS-I) and the participants (GAIS-P). The pore grading criteria used for clinical evaluation are summarized in Table 2.

**Table 2:** Pore grading criteria used for clinical assessment.

Grade	Classification	Description
5	Very large pores	Prominent, clearly visible pores with irregular surface texture; noticeable even at conversational distance
4	Large pores	Easily visible pores, mainly concentrated in the T-zone and malar areas; mild irregularity of surface texture
3	Moderate pores	Visible upon close inspection; overall skin texture moderately refined with mild unevenness
2	Small pores	Minimally visible pores; smooth surface texture with good light reflection and uniformity
1	Very small or invisible pores	Pores virtually imperceptible; smooth, uniform skin texture with high surface regularity

### Data analysis

Because of the pilot nature of the study and small sample size, data were analyzed descriptively. Primary outcomes were changes in facial pore appearance and skin quality over time. Secondary outcomes included investigator- and patient-reported aesthetic improvement. Trends were evaluated across Days 0, 30, 60, 120, and 180 to determine the peak response period and the onset of regression in pore refinement.

## Results

### Pore size changes over time

#### Day 30

All participants demonstrated visible reduction in facial pore prominence accompanied by improved skin texture and luminosity. Both clinical evaluation and standardized photographic comparison confirmed an early treatment response following the second HCC session.

#### Day 120

**Fig. 1:**
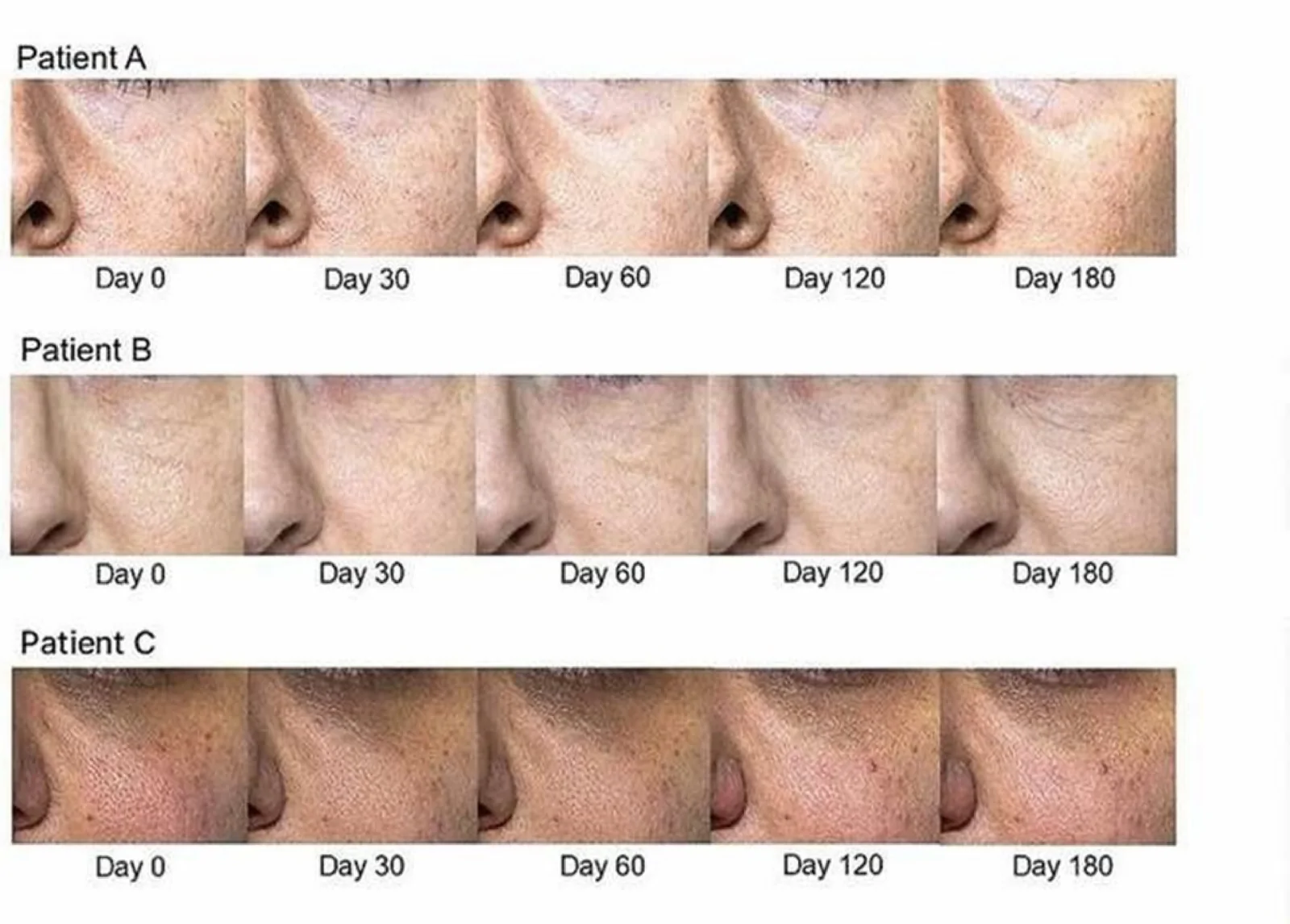
Standardized facial photographs at baseline and post-treatment showing visible pore reduction following Hybrid Cooperative Complex treatment.

**Fig. 2:**
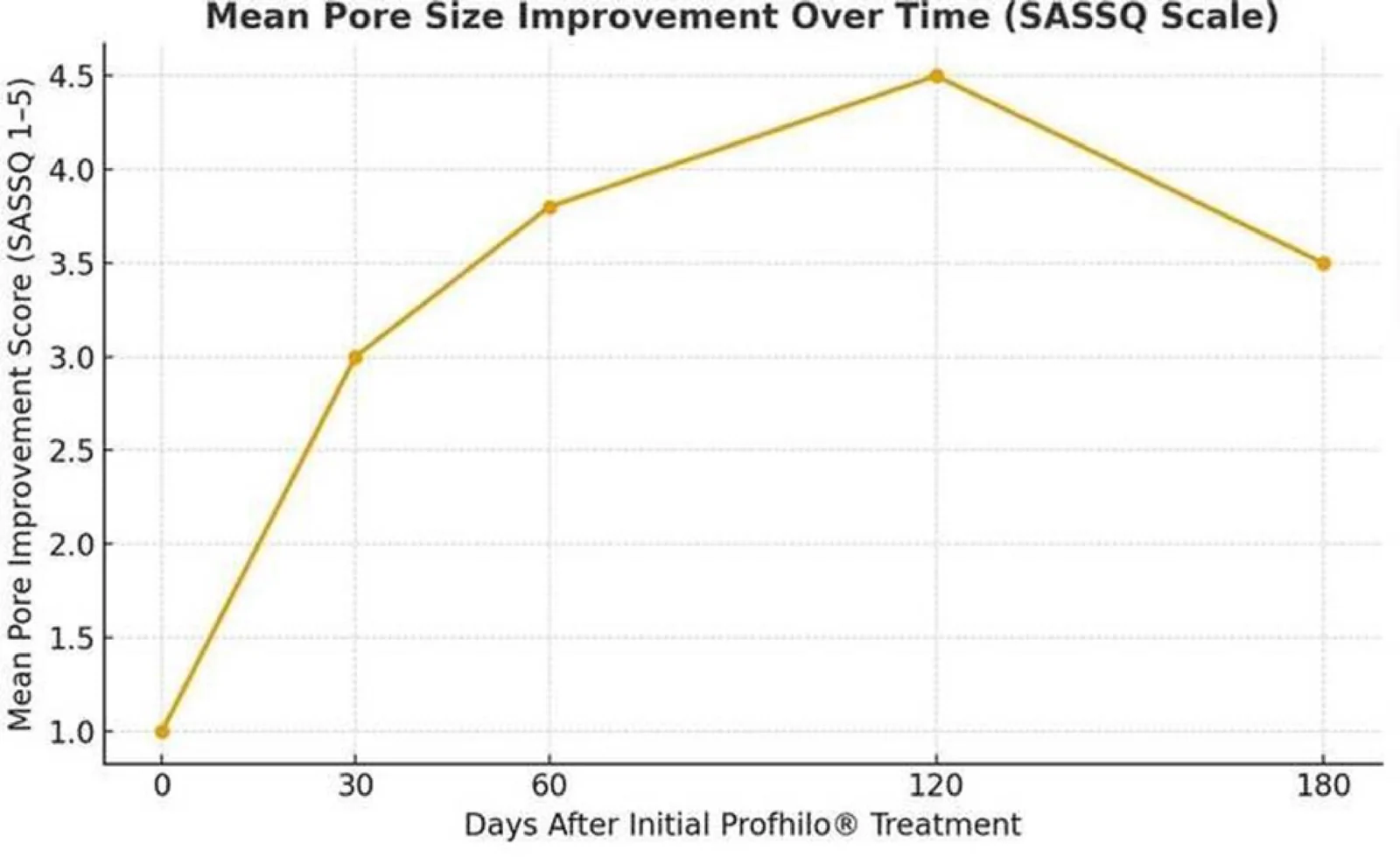
Mean facial pore size measurements before and after treatment demonstrating reduction in pore dimensions.

Peak pore refinement was observed at Day 120 in nine of ten participants, based on standardized photography and SASSQ scoring. The skin exhibited maximum smoothness, minimized pore visibility, and enhanced uniformity of surface texture during this period. Representative standardized facial photographs illustrate visible pore reduction (Fig. 1), and mean facial pore size measurements before and after treatment demonstrate a corresponding decrease in pore dimensions (Fig. 2).

#### Day 180

By Day 180, although patients and clinicians continued to report a subjectively favorable overall appearance, photographic analysis revealed regression of peak pore refinement in most cases. Despite this decline from peak response, all participants maintained skin quality that appeared improved compared with baseline. One of the ten participants, as verified by high-resolution photography and SASSQ scoring, showed persistent peak pore refinement at Day 180.

A summary of peak pore improvement and suggested maintenance timing is presented in Table 3.

**Table 3:** Summary of peak pore improvement and suggested maintenance timing. A supporting study website can be found at www.porestudy.com.

Patient ID	Age, years	Peak pore improvement, days	Regression of peak response at Day 180	Suggested maintenance timing
P01	42	120	Marked	Between Day 120 and Day 180
P02	34	120	Partial	Between Day 120 and Day 180
P03	37	180	None	Day 180 or later
P04	32	120	Marked	Between Day 120 and Day 180
P05	51	120	Marked	Between Day 120 and Day 180
P06	47	120	Marked	Between Day 120 and Day 180
P07	36	120	Partial	Between Day 120 and Day 180
P08	44	120	Marked	Between Day 120 and Day 180
P09	32	120	Marked	Between Day 120 and Day 180
P10	52	120	Marked	Between Day 120 and Day 180

## Discussion

### Clinical relevance of pore size

HCC treatment was associated with visible improvement in facial pore appearance, potentially through enhanced dermal hydration, improved dermal support, and extracellular matrix remodeling. The observed changes over follow-up suggest that pore morphology may reflect aspects of dermal remodeling status. These findings support the potential role of pore assessment as a practical clinical marker when considering maintenance treatment timing.

This study has several limitations, including the small sample size, the absence of a control group, and the descriptive nature of the analysis. In addition, reliance on photographic and clinical assessment may introduce observer bias, and no instrumental pore-measurement system was used. Larger controlled studies using objective imaging and quantitative assessment tools are needed to validate these preliminary findings.

The observed reduction in facial pore prominence following HCC treatment may be explained by multi-layered dermal remodeling mechanisms, as described in previous studies of hybrid complexes of high- and low-molecular-weight hyaluronic acid^9^. Potential mechanisms include fibroblast activation, collagen stimulation, elastin remodeling, enhanced dermal hydration, increased tissue turgor, and extracellular matrix reorganization.

These mechanisms are further supported by ex vivo and in vitro evidence from Stellavato et al., who showed that hybrid cooperative complexes may reactivate quiescent fibroblasts and promote extracellular matrix renewal^4^. Similarly, prior clinical experience with the BAP injection technique has reported improvement in skin turgor and smoothness, aligning with the present study’s findings of progressive pore refinement and improved surface uniformity^3^.

Taken together, these findings suggest that the effect of HCC may extend beyond hydration to include cellular reactivation, extracellular matrix remodeling, and surface-level texture improvement that may be reflected in pore morphology. However, these mechanisms remain inferential in the present study. Future studies using high-frequency ultrasound, optical coherence tomography, VISIA analysis, PRIMOS 3D surface analysis, or biopsy-based morphometric assessment are warranted to confirm the cellular and extracellular matrix-level changes underlying pore size modulation.

The correlation between pore size and perceived skin quality reported by Kim et al. also supports the clinical relevance of this outcome measure. Because pore volume has been shown to correlate strongly with visual assessment, reduction in pore prominence may represent both a biologically relevant and patient-perceived aesthetic endpoint^5^.

### Timing of peak effect and decline

This study identified Day 120 as the peak improvement interval for nine of ten participants, with one participant exhibiting continued improvement through Day 180. By the end of the 180-day observation period, nine of ten participants showed some degree of regression from peak pore refinement, despite maintaining a clinically favorable appearance and patient-reported satisfaction. These findings suggest that HCC-associated pore refinement may follow a temporal pattern characterized by progressive improvement, peak response, and subsequent partial decline.

### Toward pore-based maintenance timing

The observed relationship between visible pore regression and declining treatment effect suggests that pore morphology may have value as a practical, patient-observable clinical marker for estimating maintenance treatment timing. Rather than adhering strictly to calendar-based intervals, clinicians may consider re-treatment when patients or practitioners notice progressive pore re-enlargement, supported by standardized photographic assessment.

While this pilot cohort was not powered to establish definitive or age-stratified recommendations, the results underscore the potential of pore-based evaluation as a personalized, outcome-driven tool for aesthetic maintenance planning. Future randomized controlled trials with larger cohorts, comparator groups, objective measurement devices, and formal statistical testing are required to confirm these preliminary observations.

## Conclusion

This pilot study suggests that facial pore size may visibly improve following HCC treatment, with the greatest apparent effect observed around Day 120 in most participants. By Day 180, photographic evaluation indicated regression of peak pore refinement in many participants, suggesting that visible pore morphology may be useful as a practical clinical indicator when considering maintenance treatment timing.

These findings should be interpreted cautiously. The small sample size, absence of a control group, descriptive analysis, and lack of instrumental pore measurements limit generalizability and preclude definitive causal conclusions.

Future research should focus on the 120- to 180-day interval to better characterize the transition between peak response and decline and to refine individualized maintenance strategies. Objective imaging techniques, such as high-frequency ultrasound, VISIA, or PRIMOS 3D surface analysis, may help clarify the dermal and extracellular matrix-level mechanisms underlying pore refinement. Larger randomized controlled studies are required to confirm these preliminary observations.

## Data Availability

The datasets generated and/or analysed during the current study are available from the corresponding author on reasonable request.
